# Implementation science research to understand the readiness of a mobile clinic intervention to screen for high-risk HPV infections and associated morbidity in Mali and Nigeria

**DOI:** 10.52872/001c.134069

**Published:** 2025-04-17

**Authors:** Kate Klein, Imran Morhason-Bello, Lifang Hou, Mamoudou Maiga, Ibrahima Téguété, Caryn E. Peterson

**Affiliations:** 1 Havey Instititute for Global Health, Northwestern University; 2 Department of Obstetrics and Gynecology, and HPV Consortium, College of Medicine, University of Ibadan; 3 Department of Preventive Medicine, Northwestern University; 4 Department of Preventative Medicine, Northwestern University; 5 Obstetrics and Gynocology, USTTB; 6 School of Public Health, Division of Epidemiology and Biostatistics, Center for Global Health, University of Illinois Chicago

**Keywords:** HPV, Cervical Cancer, Self Sampling, Mobile Clinic, HPV DNA testing, LMIC

## Abstract

Access to and community acceptance of point-of-care testing for high-risk human papillomavirus (hrHPV) are vital to cervical cancer (CC) prevention. The World Health Organization (WHO) reports high rates of HPV-16/18 (oncogenic hrHPV strains) in Sub-Saharan Africa (SSA). In rural Mali and peri-urban Nigeria, prevention efforts are limited by cognitive, socio-cultural, logistical, and resource-related barriers, leading to ongoing transmission, late diagnoses, limited treatment options, and preventable deaths. To address these barriers, we prepared a mobile clinic intervention providing self-sample collection and rapid hrHPV testing. Pre-implementation research assessed factors influencing success in both countries. Using implementation science methods, focus groups per target community were conducted with political/administrative, religious, business, community leaders, and women of screening age. Participants also completed a short survey on HPV knowledge. Data were analysed using descriptive statistics and thematic analysis. From January 2024 to January 2025, planning occurred alongside the mobile clinic’s manufacturing and shipping. Eighteen focus groups were held between January and March 2025 - ten in Mali (with additional sessions for marketplace leaders) and eight in Nigeria. Results will inform site-specific adaptations. A follow-up study will document real-time implementation adjustments to support scale-up. Culturally tailored, context-specific strategies are key to addressing disparities in HPV-related cancer prevention. Implementation science can assess community readiness and guide effective intervention delivery.

Cancer preventive services and care in low- and middle-income countries (LMICs) has multiple challenges that hinder its success relative to many high-income countries. Some of these challenges include financial constraints, shortage of manpower (oncologists, nurses, social health workers, researchers and scientists), lack of infrastructure, diagnostic equipment and specialized training, resulting in disparities in in access to cancer preventions and care.^1,2^ Furthermore, there is a lack of access to vaccines and essential cancer pharmaceuticals challenging prevention and treatment.

Social challenges, such as low levels of public awareness about cancer prevention, risk factors, and early signs of cancer have been associated with late diagnoses and lower survival rates.^1^ Cultural beliefs and social and religious stigma can also prevent individuals from seeking preventive care and early treatment.^3^ There is a need to increase public awareness to empower communities to engage with prevention efforts. Unfortunately, public health education campaigns are often underfunded and not widespread enough to reach all populations, particularly, in LMICs.^1^

This paper details one intervention aimed at addressing these challenges, and the associated assessment of community readiness using methods derived from the field of implementation science. The intervention, a mobile clinic equipped to test and treat women for high-risk human papillomavirus (hrHPV) infection, was targeted for rural Mali and peri-urban Nigeria. These countries are where the investigators have long term partnerships in education and research, and where there are fewer preventive and clinical services available. Prior to the intervention launch, in April 2025, investigators undertook a study of community readiness, specifically the readiness levels of high level leaders and women in the community, using a mixed methods approach.

In Mali, at last estimate the cervical cancer age standardized morality rate is 28.3% making it the second most common cancer, and the leading cause of cancer related deaths in women aged 15–44.^4^ In Nigeria, at last estimate, the age standardized mortality rate was 13.2%, also the second most common cancer and the leading cause of cancer-related deaths for the same age range.^5^ While Mali has not yet introduced HPV vaccination, Nigeria introduced the single-dose HPV vaccine in October 2023, targeting girls aged 9 to 14 years old, and vaccinated 12.3 millions girls.^6^ Other preventive services such as HPV vaccination of boys, screening with HPV DNA, or cytology (Pap smear) for women are less common in both countries.

The intervention was developed to address the health systems barriers of physical distance to tertiary healthcare and laboratory testing equipment. The mobile clinic, a large automobile, was built with separate rooms for self-sample collection, a lab for sample testing using the Atila Screenfire rapid HPV DNA test, and an examination room for visual inspection with acetic acid, hand-held colposcopy and thermal ablation or Loop Electrosurgical Excision Procedure (LEEP) for those with visible lesions/abnormality in the cervix. The goal of the intervention was to increase the number of women being screened for HPV infection, and particularly to detect those with hrHPV, and engage them in further screening and treatment as necessary. The intervention was designed to reach women in the high-risk age bracket of 25–65 years old^7^ and offered at no cost.

The mobile clinic space was designed so that a participant would first collect a cervical mucus specimen via a swab inserted into the vagina.^8^ Next, this sample would be analyzed using nucleic acid amplification and the results would be interpreted by a lab technician. In most cases, women would receive their results within 60 minutes, and if needed, treatment on the same day. The three innovations used - a mobile clinic, self-sample collection, and rapid testing have all been used in other LMICs, but to the study team’s knowledge, not together in one intervention and not in rural Mali and peri-urban Nigeria.^9^

Mobile clinic interventions have been used in some countries in SSA, such as South Africa^10^ and Mozambique,^11^ with success. In SSA, bringing clinical care services into the communities of the target population could mitigate many barriers, such as, eliminating travel time and cost, reduce lost time from work, increase community awareness and knowledge about HPV and CC, assure same day results and treatment, when indicated, thereby reducing loss to follow-up.^12^

Meenan et al^12^ have shown that self-sampling for HPV (in their study with mailed HPV self-sampling kits) was a cost-effective method to increase screening numbers versus asking women to come into a traditional clinic setting. Joseph et al^13^ have also demonstrated, in a study of five African countries, that the cost of self-sampling nucleic acid amplification tests was just $9–$12 per test which was less than a Pap test conducted by a clinician. Finally, a comparison of cervical screening strategies in China against the outcomes of such indicators as years of life saved (YLS) and quality adjusted life years (QALY) showed that HPV DNA testing (for strains 16 and 18) was the most effective amongst all screening strategies in increasing YLS and QALY, if conducted every three years.^14^ Strong scientific evidence supports the use of self-sampled (SS) specimens, which yield comparable HPV detection and genotyping results to clinician-collected specimens and overcomes many barriers to testing (e.g., removes the need for a vaginal exam, overcomes concerns about male clinicians conducting exam, saves time (e.g., SS takes 2 minutes) compared to an exam) and are cost-effective making SS HPV DNA testing potentially highly feasible in SSA.^7,9,11^

The sites targeted for the mobile clinic intervention were Kita and Sikasso in Mali, and Kajola and Iseyin local government areas in the North Senatorial District, Oyo State, and the Ibadan North and NorthEast local government areas, Oyo State in Nigeria. These sites were chosen because of the existing connections of the study team to these areas, as well as their lack of regular preventive services. These sites share some similarities, but it is important to understand their unique characteristics in the design of the intervention.

Mali: Kita’s population at last census (2018) was 463,787, while in 2020, the urban area of Kita counted 65,908. The primary employment is farming and processing related to the peanut and cotton industries. The town also sits on a transit hub between Bamako and Kayes, which includes a railway station on the Dakar-Niger Railway line. The people in this area are predominantly Muslim and Catholic.^15^ Sikasso is a larger city with about 226,000 residents and is considered the second largest city in Mali after Bamako. The region is also known for farming cotton and peanuts and has a large and active marketplace. Both Kita and Sikasso are the capitals of their Commune, or local government area. Commune affairs are directed by a commune council of elected members and a commune executive of the elected mayor.^16^ The majority of healthcare in Mali is provided for by Non-Governmental Organizations, and the health system is decentralized. There is a country level Ministry of Health and Welfare, and regional hospitals.^17^

Nigeria: Kajola and Iseyin local government areas are considered more peri urban located outside the major city of Ibadan. At the last census, Kajola had a population of 318,000 and Iseyin had a population of 405,500. Ibadan North had a population of 488,725 and Ibadan NorthEast had a population of 525,725. The people in this area predominantly speak Yoruba and English, and are Muslim, Christian, and local religions. The state is known for farming crops such as cassava cocoa and tobacco.^18^ The Ministry of Health coordinates policy and programs for the entire state. The University of Ibadan and University College Hospital are premier federal government owned tertiary institutions. The state government has its own tertiary, secondary and primary healthcare facilities.^19^

## METHODS

As the intervention was targeted for communities who had not had exposure to this type of intervention, the study team felt it was important to understand the readiness determinants (i.e. the facilitators and barriers) that may come into play from influential sources such as cultural, religious, political/administrative, and market/community leaders who may support or oppose this intervention and would potentially work with/for or against the implementation of this intervention. Additionally, the researchers felt it was important to understand the viewpoints of women in the target demographic, women in the community, who are both influenced by community leadership, as well as influenced by their peers. From January 2024 to January 2025, this implementation science focused study was planned, alongside the manufacturing and shipping of the vehicle for the mobile clinic, and the initial planning for the implementation of the intervention.

The Consolidated Framework for Implementation Research (CFIR) provided a useful conceptual model to assess readiness for implementation. Within the CFIR, the research was focused in the domain of the Inner Setting, utilizing a variety of subconstructs such as; compatibility, leadership engagement, culture, and relative priority to frame the development of the study tool which aimed to characterize the local determinants (facilitators and barriers) as defined by different groups in the community. The CFIR defines, ‘high level leaders’ and ‘innovation’ recipients as important members of the community in terms of their levels of influence and agency to make choices for themselves and others. Within the CFIR, high level leaders and individuals are at the top and bottom of the hierarchy, respectively. High level leaders are defined as, “individuals with a high level of authority, including key decision-makers, executive leaders, or directors.” ‘Innovation recipients’ are defined as “individuals who are directly or indirectly receiving the innovation.” These people will make individual choices, and they are likely to influence their peers through their decisions.^20^

Weiner et al define readiness as the level to which community members would “act or respond to support change implementation,” along the continuum of reluctance, on the one hand, to complete readiness. For this intervention the readiness constructs of “openness and receptivity” within individual readiness^20^ were targeted for exploration. Openness and receptivity have been defined as “overall positive or negative evaluative judgment of a change initiative”.^21^

To understand readiness amongst leaders and women targeted for the intervention, the team planned focus groups, one for each subgroup, in which participants would be asked about their knowledge and beliefs about HPV, knowledge and beliefs about the mobile clinic intervention, opinion leaders in the community, how the participants would or would not be involved in promoting the intervention, and perceptions of motivations and barriers of the target intervention community to be screened for HPV. A convenience sample was selected amongst those who responded to advertisements in the community in Mali. In Nigeria, a team from the team from Oyo State Primary Health Care Board selected participants. Each focus group consisted of no more than ten participants. Each participant was given a number to use when speaking so that no identifying information about the participant was included in the transcripts. The focus groups were moderated by a local member of the Malian and Nigeria study teams and conducted in the local language. Participants were consented using a paper consent form, as well as through verbal consent for those who were not literate.

The transcripts from the focus groups would then be transcribed and translated. The translated transcripts would then be analyzed for themes using MAXQDA qualitative software. The study team will conduct collaborative coding, with one member of the team from a high-income partner university and the other coder from the local (Malian or Nigerian) university. The coders will use both the CFIR codebook template, using code words related to readiness in the Inner Setting and with Individuals, as well as a priori codes that emerge and relate to participants’ openness and receptivity to the intervention. Taken together the emergent themes would then be used to characterize their level of readiness.

At the recruitment stage, recruiters would also capture limited demographic data, including sex, age, and job title (for the leaders) and pre-focus group knowledge of HPV, HPV connection to cervical cancer, and existence of a vaccine. Each participant responded on a Likert Scale of 1 (not important) to 5 (very important) for question around the level of importance of vaccinating and screening for HPV. Summary descriptive statistics will be calculated to determine pre focus group median and modes, standard deviations, and proportions.

Findings from the study will then be summarized and provided to the intervention planning team, with the intention of supporting the context specific adaptations at each site. A similar study will is planned to take place during the intervention period (2025–2026) to assess the level of acceptance of the intervention, and what and why adaptations are made in response to community determinants.

## RESULTS

A total of twenty-six (26) focus groups were conducted between January and March 2025, ten in Mali and sixteen in Nigeria. The Malian focus groups separated marketplace leaders from community leaders, which added two extra focus groups. In Nigeria, a total of one hundred and thirty-three (133) participants were recruited across four sites; Iseyin, Kajola, Ibadan North and Ibadan Northeast. Males who participated in the sessions were 67 (50.4%) and females were 66 (49.6%) The lowest in age was 25 years and the highest in age was 84 years with a mean age of participants of 55.1 years. In Mali, a total of ninety-nine (99) participants were recruited across two sites; Kita and Sikasso. Males who participated in the sessions were 68 (68.7%) and females were thirty-one (31.3%). The lowest age was 25 years, and the highest age was 68 years with a mean of 54.1 years.

At the time of publication, data from the focus groups is being analyzed and will be reported in subsequent manuscripts. Quantitative results will include averages for knowledge of the HPV virus, and awareness of the HPV vaccine. Median scores will be reported for Likert scale questions around importance of HPV vaccination and importance of HPV treatment. Collaborative coding between researchers in the United States, Mali and Nigeria will be conducted, followed by thematic analysis. Results will be used to adapt the planned intervention activities to each specific site in Mali and Nigeria. A future study is being planned to document adaptations that happen at each site during the implementation period. This will be used to inform expansion of the intervention to other sites.

## DISCUSSION

This manuscript highlights the intention of our team to investigate the readiness of critical stakeholders before adopting a mobile clinic intervention as a strategy to improve access of women in CC prevention. According to Scheirer,^22^ five common factors identified to improve sustainability of interventions include “(a) a program can be modified over time, (b) a “champion” is present, (c) a program “fits” with its organization’s mission and procedures, (d) benefits to staff members and/or clients are readily perceived, and (e) stakeholders in other organizations provide support.” Of all these factors, support of stakeholders and end-users is critical from the preparation or planning stage for efficient implementation and sustainability of the intervention.

Community engagement provides an opportunity to learn and understand the perspectives of people who are the end-users of a public health intervention such as the mobile clinic.^22–24^ We envision that information from the proposed research will help those carrying out the intervention to better understand the key issues that might guide the implementation and sustainability of this investment.

It is equally important to understand the views of stakeholders on their strategies to sustain this intervention after the expiration of the current stream of funding. Sustainable planning will cover maintenance of infrastructure, consumables and manpower to ensure smooth running of this intervention. Mobile clinics, as with all vehicles, require funds for maintenance, especially in conditions where there are unpaved roads and extreme flooding and heat among other environmental challenges common to both Nigeria and Mali. Degradation of mobile clinic and equipment is a risk to our proposed intervention, and it is essential for long-term sustainability plan. From an economic perspective, sustainability of the intervention will rely on continuous funding sources. It is therefore crucial to involve political/administrative leaders from the outset to ensure they understand the significance of the program and foster long-term local support. However, Perkins and Hornak^25^ problematize characterizing sustainability as linear, in that it comes as the last step or stage of implementation. They support a dynamic model which, “considers adaptations or modifications in the implementation process, as well as the context and system interventions. It supports building adaptive capacity for the implementing bodies to make changes based on need.” A more dynamic definition of sustainability allows for a more holistic consideration of understanding which parts of an intervention sustain over time. Inevitably, there will be changes of the demographics of communities, the health systems that serve those communities, HPV and cervical cancer screening technologies, access to vaccines amongst others. Shelton and Lee^26^ argued that determination of sustainability should not be assessed from a binary response of “yes/no” because of several multifaceted considerations that affects it. Some of the factors that influence sustainability include continuing or improving health benefits, maintaining community or organizational partnerships (e.g., coalitions) and capacity, and continuing intervention activities or core components (determined a priori).” Future studies will include these more holistic approaches to evaluating sustainability.

The mobile clinic intervention aims to serve as a supportive strategy to fast-track elimination of CC in Nigeria and Mali by promoting and providing opportunity to access screening for CC. Community engagement of political, cultural, religious, and community leaders may provide a platform to domesticate CC prevention strategies. The methods used to understand readiness for this intervention may support other government and multilateral organizations in CC prevention space.

## CONCLUSIONS

This manuscript provides some insights into how to incorporate implementation science methodologies prior to an intervention to characterize and contextualize determinants of readiness and acceptability in distinct communities. The information that will be collected from stakeholders will assist in designing a context driven and sustainable implementation strategy to maintain the mobile clinic as a complimentary intervention to reduce disparities in accessing cancer prevention care in people who may be hitherto unlikely to benefit from this specialized service.
